# Sex Differences in Obesity and Cognitive Function in Chinese Elderly Patients With Chronic Schizophrenia

**DOI:** 10.3389/fendo.2022.742474

**Published:** 2022-04-01

**Authors:** Wei Li, Sun Lin, Ling Yue, Yuan Fang, Shifu Xiao

**Affiliations:** ^1^ Department of Geriatric Psychiatry, Shanghai Mental Health Center, Shanghai Jiao Tong University School of Medicine, Shanghai, China; ^2^ Alzheimer’s Disease and Related Disorders Center, Shanghai Jiao Tong University, Shanghai, China

**Keywords:** elderly, obesity, schizophrenia, Chinese, cognitive impairment, sex difference

## Abstract

**Background:**

It is well known that schizophrenia is associated with sex differences. However, no study has explored the sex differences in obesity and cognitive function in elderly Chinese patients with schizophrenia.

**Objective:**

This study aimed to compare sex differences in obesity and cognitive function in elderly Chinese individuals with schizophrenia.

**Methods:**

A total of 304 elderly patients with schizophrenia and 130 sex- and age-matched healthy controls from the community were recruited. Demographic, clinical, and lipid parameters were collected for all subjects. The Montreal Cognitive Assessment (MoCA) was used to assess the global cognitive functions of the participants, while the Positive and Negative Syndrome Scale (PANSS) was used to assess psychopathological symptoms in patients with schizophrenia.

**Results:**

Of the patients with schizophrenia, the prevalence of obesity in men and women was 11.7% (19/163) and 21.3% (30/141), respectively. The score (14.51 ± 6.504) of MOCA in elderly male patients with schizophrenia was significantly higher than that (11.40 ± 6.822) in female patients. There was a positive correlation between the MOCA scores and body mass index (BMI) (r=0.206, p=0.018) in male elderly patients with schizophrenia. Conversely, the MOCA scores of female elderly patients with schizophrenia did not correlate with BMI (p>0.05). However, we found no sex differences in obesity and cognition among control older adults.

**Conclusions:**

Our findings suggest that there are significant sex differences in obesity and cognitive function in elderly Chinese patients with schizophrenia.

## Introduction

Sex differences have been reported in schizophrenia (1). Previous studies have shown that the incidence rates (cases per 100,000 per year) of schizophrenia in men are 1.4 higher than in women (2). Moreover, men with schizophrenia typically present with more negative symptoms (e.g. Flattened affect, social withdrawal) (3), and they are more likely to have fewer years of education, are less productive at work, and have more impaired social functioning than women (4). In contrast, women tend to have a later onset (on average, women develop the disease three to four years later than men, with a second peak around menopause (5)) and fewer symptoms. Additionally, since women are less likely to suffer from social drift, substance abuse, and law infringement, they often have a better course and outcome (6).

Cognitive impairment is another prominent clinical feature of schizophrenia (7). Patients with schizophrenia often show cognitive deficits across several domains, including attention, memory, learning, executive functioning, and cognitive processing speed (8). They have been linked to poor functional outcomes and long-term disability (9). Sex differences in cognition are also found in patients with schizophrenia; for example, men often show an advantage in visuospatial abilities, whereas women show an advantage in verbal fluency, visual scanning, verbal memory, and fine motor skills (10). Moreover, executive functioning may have a lesser impact on female patients’ symptoms and function profiles with schizophrenia spectrum than on male patients (11). Moreover, men with schizophrenia tend to have more significant overall cognitive deficits (12). However, studies have shown no differences in cognitive performance between men and women with schizophrenia (13). Therefore, the conclusions of relevant studies are not consistent, and we speculate that they may be related to the inclusion criteria, assessment tools, and the effects of drugs.

Obesity is another critical issue in patients with schizophrenia (14) due to sedentary lifestyles, side effects of antipsychotics, and poor dietary choices resulting from cognitive deficits (15). Obesity is linked to greater morbidity, mortality, and decreased life expectancy and lower quality of life (16–18). The connection between obesity and worse cognitive performance has been demonstrated in non-psychiatric samples (19–21). Some studies have shown that obesity can also increase cognitive impairment in individuals with schizophrenia. For example, Lindenmayer et al. (22) reported a significant association between waist circumference and domains of attention/vigilance and processing speed, suggesting that obesity might lead to cognitive impairment. Spangaro et al. (23) found that elevated body mass index (BMI) might contribute to white matter (WM) disruption in schizophrenia by hampering structural connectivity in critical cortico-limbic networks. However, some studies show that cognition is not associated with obesity (24, 25). Therefore, pertinent research conclusions are inconsistent.

Over the past few decades, the interest in differences between men and women with schizophrenia has expanded to obesity and neurocognitive function (26). For example, Fang Yang et al. found that there were sex differences in obesity, BMI, and brain-derived neurotrophic factor (BDNF) levels, and BMI was only negatively correlated with BDNF in female patients (27). Xiao-E Lang et al. found sex differences in white matter dis-connectivity and its relationship to psychopathological symptoms in an early course of schizophrenia onset (4). Moreover, Xinxin Huang et al. pointed out that miR-195 was associated with cognitive impairment in female schizophrenia patients, and it may be involved in the underlying mechanism of sex differences in cognitive impairment in schizophrenia (28). Understanding these sex differences can help better understand the pathogenesis of schizophrenia and give more targeted treatment to patients of different genders. Genes have important influences on obesity and cognition, and both obesity and cognitive levels will change with age (29, 30). However, to the best of our knowledge, there is no research on sex differences in obesity and cognitive function in elderly Chinese patients with schizophrenia. This is therefore the first (cross-sectional) study to explore the effects of sex on obesity and cognitive function in elderly patients with schizophrenia.

## Materials and Methods

### Sample Size Calculation Basis

Previous studies suggested that the prevalence of obesity in chronic schizophrenia was 16.4% (31), and the prevalence of obesity in male and female patients with schizophrenia was 31.82% and 15.83% (32), respectively. Therefore, at least 30/0.32 = 93.75 for males and 30/0.16 = 187.5 for females were required for estimation based on the minimum sample size of 30 for each group, and a total of at least 282 subjects were included. Finally, 304 elderly patients with schizophrenia were included.

### Participants

#### Inclusion and Exclusion Criteria

Based on the previous sample size calculation, a total of 304 elderly patients (men/women=163/141) with schizophrenia were recruited from the Shanghai Mental Health Center. All participants were required to meet the following requirements: 1) age ≥ 60 years; 2) diagnosed with schizophrenia according to the Diagnostic and Statistical Manual of Mental Disorders (DSM-5): Two (or more) of the following: 1) delusions; 2) hallucinations; 3) disorganized speech; 4) grossly disorganized or catatonic behavior; 5) Negative symptoms, i.e., affective flattening, alogia, or avolition; and each present for a significant portion of time during a 1-month period (or less if successfully treated); 3) without severe medical conditions, such as cancer and infections; and 4) willingness to cooperate. Subjects with schizoaffective disorder, depressive or bipolar disorder with psychotic features, cancer, dementia, cardiovascular disease, organic brain disease, sex hormone use history or with some relevant diseases of sex hormone disorder were ruled out. All the eligible participants’ information, including general demographic information (age, education, sex), daily life habit information (smoking, drinking, drinking tea, physical exercise, and hobbies), and currently prescribed medicines (clozapine, olanzapine, quetiapine, risperidone, aripiprazole), was collected using standardized questionnaires. Additional information was gathered from medical records and collateral resources. A complete physical examination, medical history, and laboratory tests were also performed for each subject. Detailed inclusion criteria and information collection process were introduced in our previous studies (33).

Moreover, 130 healthy controls (60 males and 70 females) were also recruited by advertisements at the local community, who were matched for age and sex with patients with schizophrenia (they had a mean education of 10.60 ± 3.528 years and an average age of 69.55 ± 7.817 years). All healthy controls were interviewed by trained investigators overseen by a research psychiatrist. None had any personal or family history, nor any psychiatric evaluation for clinical mental illness.

#### Ethical

The Research Ethical Committee of the Affiliated Mental Health Center of Shanghai Jiaotong University School of Medicine approved the study protocol. Written informed consent was obtained from all the participants before the study. All research procedures were carried out per the principles of the Declaration of Helsinki.

### Measurement of Body Mass Index (BMI), Fasting Blood Glucose, and Lipid Profile

BMI was determined by dividing weight by height squared (kg/m^2^). Based on the Chinese Working Group on Obesity in China (WGOC) criteria (34), participants with BMI≥28 were classified as obese and those with BMI < 28 as non-obese. Peripheral blood samples were collected between 7 and 9 am after an overnight shift. The values of serum fasting blood glucose, triglyceride, cholesterol, low-density lipoprotein, and high-density lipoprotein were obtained using the hexokinase method on an auto-analyser (Dimension Xpand plus).

### Cognitive Assessment and Psychiatric Symptom Assessment

The Montreal Cognitive Assessment (MoCA) (35) and the Positive and Negative Syndrome Scale (PANSS) (36) were used to assess the overall cognitive function and the severity of psychotic symptoms of the participants, respectively. Previous studies have suggested that MoCA is a validated, clinician friendly, brief instrument for screening cognitive deficits in schizophrenia (37, 38). Moreover, we also utilized the Geriatric Depression Scale (GDS) (39) to exclude depression, and a GDS score of 10 or more was considered depressed. In our current study, no one was excluded because of depression.

### Statistical Analysis

Continuous variables are expressed as mean ± standard deviation or median (p25, p75) and categorical variables as frequencies (%). First, a single-sample Kolmogorov-Smirnov test was used to check whether the data was normal. Next, an independent sample t-test was used to compare the normal data between the male and female groups. The Mann-Whitney U test was used to compare data with a non-normal distribution. The Chi-square test was used to analyze the categorical variables between the two groups. The MoCA was analyzed using a 2 × 2 ANOVA representing the factors of obesity and gender (male vs. female). In the MoCA comparisons, age and education level were used as covariables in the multivariate analysis of covariance (MANCOVA). They were also included in the MoCA total score and its seven cognitive domains of dependency measures to examine significant diagnostic differences. The independent predictors were group, gender, and group×gender interaction. In the *post hoc* comparisons, a multiple testing correction was also performed. After controlling for several potential confounding factors, such as age, education level, and clinical variables, partial correlation analysis was used to explore the relationship between MOCA and BMI. Two-tailed tests were performed at a significance level of P<0.05, and Bonferroni correction was used to correct p-values for multiple comparisons. All the statistical analysis was performed using SPSS version 22.0 (IBM Corporation, Armonk, NY, USA).

## Results

### Prevalence of Obesity in Men and Women in Patients With Schizophrenia and Controls

Of the patients with schizophrenia, the prevalence of obesity in men and women was 11.7% (19/163) and 21.3% (30/141), respectively. The Chi-square test results showed a statistically significant difference (p=0.028) in the prevalence of obesity in men and women. In control older adults, the prevalence of obesity was 6.7%(4/60) in men and 15.7%(11/70) in women. However, chi-square results showed no difference (p=0.168) between the two groups.

### Sample Characteristics

Demographic and clinical characteristics of schizophrenia are summarized in **Table 1**. There were significant gender differences (p<0.05) in age, duration of disease, age of onset, fasting blood glucose, cholesterol, high density lipoprotein, low density lipoprotein, PANSS total, PANSS positive symptoms, PANSS general psychopathology, diabetes, smoker, drinker, and tea drinker. However, there were no significant gender differences (p>0.05) in education, triglyceride, GDS, PANSS negative, hypertension, hyperlipidemia, physical exercise, hobby, clozapine, olanzapine, quetiapine, risperidone and Aripiprazole. There was a significant interaction between obesity and gender in age, education, duration of disease, age of onset, fasting blood glucose, cholesterol, high density lipoprotein, low density lipoprotein, PANSS total, PANSS positive symptoms, PANSS general psychopathology, diabetes, smoker, drinker, and tea drinker (p < 0.05). Moreover, there were no significant gender differences (p>0.05) in age, education, BMI and MoCA in control older adults. **Table 2** shows the results.

**Table 1:** Demographic and clinical data in schizophrenia with and without obesity.

Variables	Schizophrenia with obesity		Schizophrenia without obesity		Group	Gender	Group × Gender
	Male (n=19)	Female (n=30)	Male (n=144)	Female (n=111)	F (p value)	F (p value)	F (p value)
Age, y	63 (63,66)	66.5 (62.8,75.0)	65 (62,69)	66 (62, 72)	0.307 (0.759)	-2.818 (0.005*)	2.781 (0.041*)
Education,y	7 (4,9)	9 (6,10.3)	9 (7, 9)	9 (4,10)	-1.303 (0.194)	0.711 (0.477)	2.726 (0.044*)
Duration of disease, y	36 (28,46)	38 (20.8, 45.0)	41 (32.8, 46)	38 (23,44)	-0.845 (0.399)	2.663 (0.008*)	2.843 (0.038*)
Age of onset,y	30 (17,38)	30.5 (22.0, 48.0)	24 (20, 32)	32 (24,43)	1.032 (0.303)	-4.220 (<0.001*)	5.969 (0.001*)
Fasting blood glucose, mmol/L	5.6 (4.9, 6.0)	5.6 (4.8, 7.1)	5.1 (4.6,5.5)	5.1 (4.6, 6.3)	2.724 (0.009*)	-2.478 (0.014*)	6.231 (<0.001*)
Triglyceride, mmol/L	1.4 (1.1, 1.9)	1.4 (1.1, 1.9)	1.0 (0.7, 1.6)	1.3 (0.9, 1.6)	1.024 (0.307)	-1.772 (0.077)	1.275 (0.283)
Cholesterol,mmol/L	4.4 (3.6, 4.7)	4.8 (4.0, 5.5)	4.4 (3.9, 5.0)	5.1 (4.3, 5.5)	-0.210 (0.834)	-4.955 (<0.001*)	8.661 (<0.001*)
High density lipoprotein,mmol/L	1.0 (0.9,1.3)	1.3 (1.1,1.5)	1.1 (1.0, 1.4)	1.3 (1.1, 1.7)	-1.142 (0.254)	-3.170 (<0.001*)	5.707 (0.001*)
Low density lipoprotein, mmol/L	2.6 (1.9,3.0)	2.9 (2.3, 3.5)	2.5 (2.1,3.2)	2.8 (2.3, 3.4)	0.988 (0.324)	-2.639 (0.009*)	3.276 (0.021*)
GDS	7.5 (7.0,16.8)	9.0 (5.5, 14.0)	9 (5, 14.0)	10.0 (6,15.3)	-0.324 (0.746)	-0.603 (0.547)	0.194 (0.901)
PANSS total	56.5 (47.5,69.3)	62.5 (52.0, 78.3)	57 (47,70)	61 (49, 83)	-0.091 (0.928)	-2.872 (0.004*)	3.019 (0.030*)
PANSS positive	7.5 (7.0,16,8)	9.5 (7.0, 14.3)	10 (8.0, 13.0)	11 (8,17)	-0.335 (0.738)	-2.432 (0.016*)	2.662 (0.049*)
PANSS negative	14.0 (11.3, 21.8)	17 (12, 25)	16.5 (13.0, 24.0)	17 (12,26)	-0.808 (0.420)	-1.504 (0.134)	1.253 (0.291)
General mental sub-score	28.5 (26.8,33.3)	33 (27, 41.3)	29 (25.3,34.0)	30 (26,40)	0.322 (0.748)	-2.939 (0.004*)	3.047 (0.029*)
Hypertension, n (%)	9 (47.4)	17 (56.7)	51 (35.4)	36 (32.4)	6.316 (0.015*)	0 (1.000)	6.985 (0.072)
Diabetes, n (%)	6 (31.6)	18 (60.0)	22 (15.3)	32 (28.8)	16.657 (<0.001*)	12.417 (0.001*)	27.617 (<0.001*)
Hyperlipidemia, n (%)	11 (57.9)	15 (50.0)	61 (42.4)	33 (29.7)	4.514 (0.039*)	3.772 (0.061)	9.003 (0.029*)
Smoker, n (%)	13 (68.4)	1 (3.3)	83 (57.6)	1 (0.9)	0.359 (0.619)	116.347 (<0.001*)	115.292 (<0.001*)
Drinker, n (%)	4 (21.1)	1 (3.3)	24 (16.7)	4 (3.6)	0.026 (1.000)	14.970 (<0.001*)	14.853 (0.002*)
Tea drinker, n (%)	8 (42.1)	2 (6.7)	38 (26.4)	17 (15.3)	0.033 (1.000)	9.305 (0.003*)	13.296 (0.004*)
physical exercise, n (%)	7 (36.8)	9 (30.0)	44 (30.6)	36 (32.4)	0.031 (0.868)	0 (1.000)	0.385 (0.943)
Hobby, n (%)	6 (31.6)	10 (33.3)	54 (37.5)	41 (36.9)	0.375 (0.628)	0.064 (0.813)	0.400 (0.940)
Clozapine, n (%)	2 (10.5)	2 (6.7)	24 (16.7)	20 (18.0)	2.555 (0.135)	0.026 (1.000)	2.772 (0.428)
Olanzapine, n (%)	3 (15.8)	12 (40.0)	34 (23.6)	33 (29.7)	0.393 (0.598)	3.313 (0.073)	5.045 (0.168)
Quetiapine, n (%)	3 (15.8)	5 (16.7)	18 (12.5)	18 (16.2)	0.162 (0.661)	0.594 (0.515)	0.869 (0.833)
Risperidone,n (%)	5 (26.3)	8 (26.7)	38 (26.4)	38 (34.2)	0.213 (0.733)	1.150 (0.314)	2.077 (0.557)
Aripiprazole,n (%)	5 (26.3)	4 (13.3)	31 (21.5)	16 (14.4)	0 (1.000)	3.445 (0.076)	3.415 (0.332)

* means p < 0.05; MoCA means Montreal Cognitive Assessment; GDS means Geriatric Depression Scale (GDS); PANSS means the Positive and Negative Syndrome Scale.

**Table 2:** Sex difference in BMI and cognitive function in normal elderly.

Variables	Male(n=60)	Female(n=70)	t	p
Age, y	70.20 ± 7.474	69.00 ± 8.112	0.872	0.385
Education, y	11.12 ± 3.335	10.16 ± 3.650	1.555	0.123
MoCA	25.68 ± 2.765	25.51 ± 3.646	0.294	0.769
BMI	23.55 ± 2.773	24.49 ± 3.793	-1.592	0.114

### Gender Difference in Cognitive Performance in Schizophrenia and Controls With and Without Obesity

**Figure 1:**
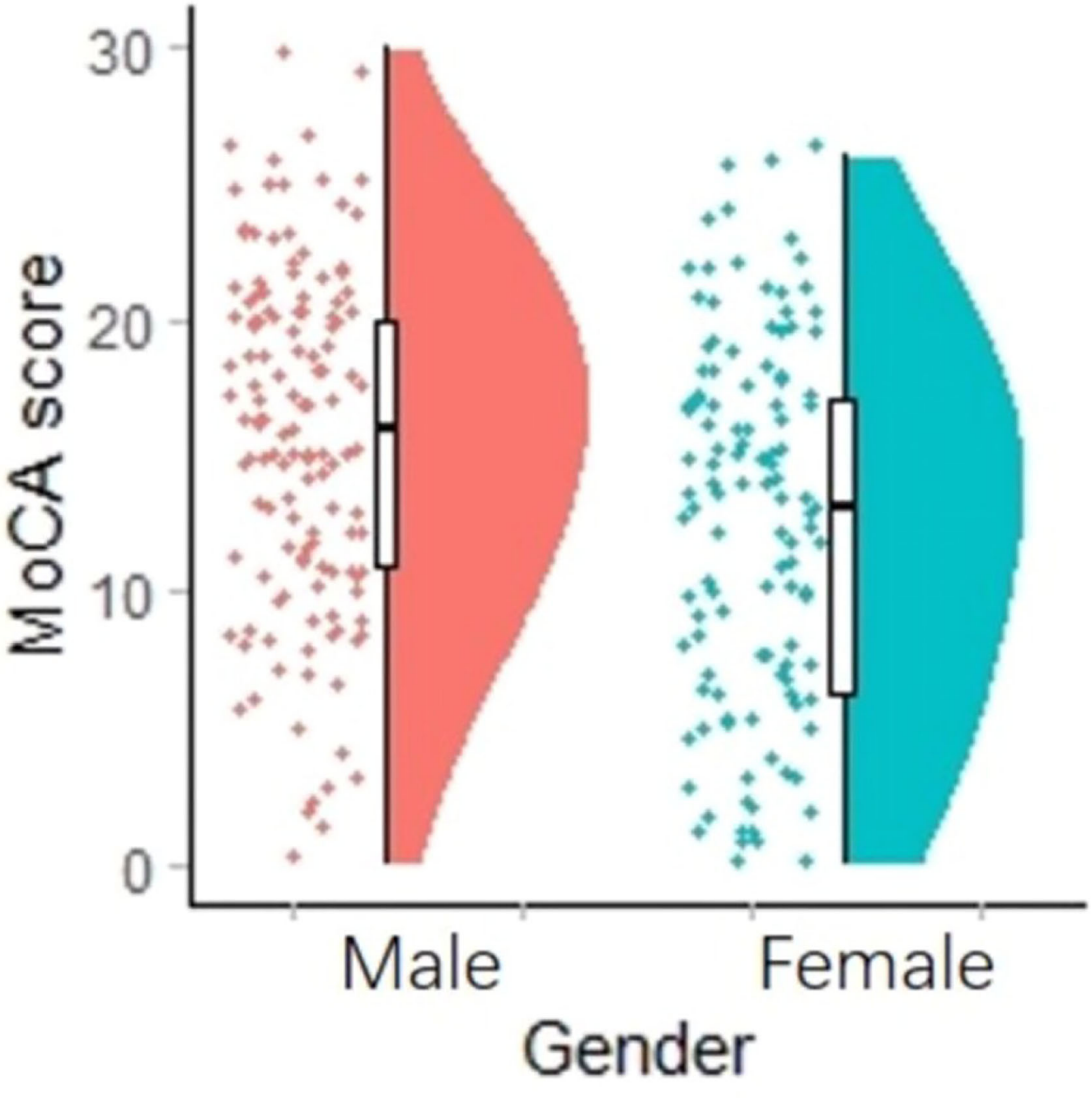
Comparison of MoCA between male and female.

Multivariate analysis of covariance (MANCOVA) was used to investigate the effects of gender and obesity on cognitive functions. After controlling for age, education and PANSS, we found no statistically significant differences in MoCA scores and subtests between obesity and non-obesity groups, while male schizophrenics had higher MoCA scores, visual space and executive function, named, attention, abstract ability, and orientation than female schizophrenics (**Figure 1**); Multivariate analysis of covariance (MANCOVA) and *post hoc* comparisons showed that gender and obesity had no interaction effect on MoCA total score and other sub-test scores (when both sex and obesity were included, gender differences in MOCA scores and subtests disappeared). **Table 3** shows the results. In control older adults, we found that there was no effect of gender or obesity on cognitive function.

**Table 3:** Cognitive function in schizophrenia with and without obesity.

Variables	Schizophrenia with obesity		Schizophrenia without obesity		Group	Gender	Group × Gender
	Male (n=19)	Female (n=30)	Male (n=144)	Female (n=111)	F (p value)	F (p value)	F (p value)
MoCA	14.0 (7,20.3)	11 (9.5, 14.5)	15 (10.0,19.0)	12 (5,17)	0.261 (0.610)	8.711 (0.003*)	0.468 (0.494)
Visual space and executive function	1 (1,2)	1 (0.3,1.0)	1 (1.0, 2.0)	1 (0, 2)	0.677 (0.411)	8.950 (0.003*)	1.073 (0.301)
Named	2 (1.5, 2.5)	1 (1,2)	2 (1.3)	1 (0, 2)	0.629 (0.429)	9.980 (0.002*)	0.929 (0.336)
Attention	1 (1,2)	1 (0,2)	1 (1,2)	1 (0, 2)	1.859 (0.174)	20.553 (<0.001*)	0.218 (0.641)
Language	0 (0,1.5)	0 (0, 1)	1 (0, 2)	0 (0, 1)	0.029 (0.864)	1.005 (0.317)	0 (0.995)
Abstract ability	1 (0,1.5)	0 (0,1)	0 (0, 1)	0 (0, 1)	0.557 (0.448)	9.330 (0.003*)	1.311 (0.253)
Delayed recall ability	0 (0,2)	0 (0.0)	0 (0, 1)	0 (0, 1)	0.020 (0.887)	2.529 (0.113)	3.482 (0.063)
Orientation	5 (2.5,6)	5 (2.5, 5.5)	5 (4,6)	4 (2,6)	0.008 (0.928)	4.103 (0.044*)	0.181 (0.671)

* means p < 0.05; MoCA means Montreal Cognitive Assessment.

### Relationship Between MoCA and BMI in Schizophrenia and Controls

**Figure 2:**
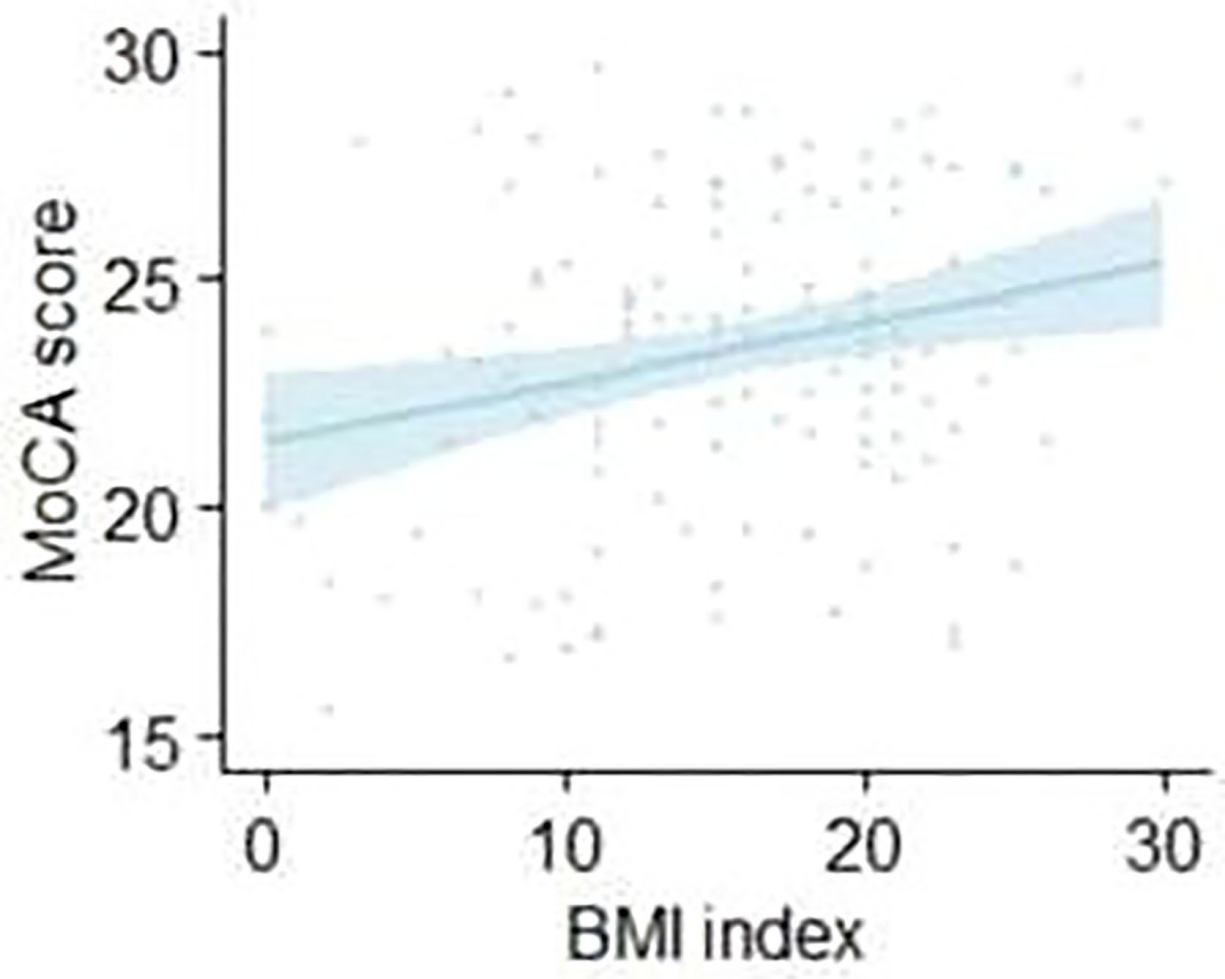
The correlation between MOCA score and BMI in male elderly patients with schizophrenia.

We then explored the relationship between BMI and cognitive functions in both sexes in patients with **schizophrenia**. Using partial correlation analysis (age, education, and PANSS were controlled), we found a positive correlation between the MOCA score and BMI (r=0.206, p=0.018) in male elderly patients with schizophrenia. Conversely, the MOCA score of female elderly patients with schizophrenia did not correlate with BMI (r=0.053, p=0.578). **Figure 2** shows the results. As above, we also did not find a link between BMI and MOCA in control older adults in both sexes.

## Discussion

This study revealed several interesting findings: 1) the prevalence of obesity in elderly Chinese men and women with schizophrenia was 11.7% and 21.3%, respectively; 2) the MOCA score in elderly male patients with schizophrenia was significantly higher than that in female patients, and there was no interaction between gender and BMI; 3) there was a positive correlation between MOCA scores and BMI (r=0.206, p=0.018) in male elderly patients with schizophrenia, while the scores of female elderly patients with schizophrenia did not correlate with BMI (p>0.05). However, this conclusion does not apply to general elderly people.

In the present study, we found that female patients had a higher prevalence of obesity than male patients (nearly double), consistent with previous studies (17, 40, 41). Moreover, compared to non-obese individuals, obese women had a higher proportion of hypertension, diabetes, and hyperlipidaemia, suggesting that they develop metabolic syndrome (42, 43). However, in our study, we did not find that obesity had any effect on mental symptoms or cognitive function among female patients with schizophrenia, inconsistent with other research conclusions (44, 45). The reason may be related to our failure to consider the effects of medication dose and menopause on antipsychotic medications whose metabolism is influenced by estrogen levels.

In addition, we found that aging men with schizophrenia had better overall cognitive function than women. The above conclusion is still valid after controlling for age, education, duration of disease, and other related variables. Zhang BH (46) reported that in schizophrenia with diabetes, men had significantly worse cognition than women in all cognitive domains. Contrastingly, in schizophrenia without diabetes, men showed worse performance in immediate and delayed memory than women. Zhang et al. (40) found that male patients with schizophrenia had more cognitive impairment than female patients in reasoning and problem solving, working memory, social cognition, verbal learning, processing speed, and visual learning. Zhang XY (8) found that male patients with chronic schizophrenia had significantly lower immediate memory and delayed memory scores than female patients. Therefore, our conclusions are the opposite. We noticed that the age of male patients (66.31 ± 5.895) was significantly lower than that of female patients (68.32 ± 7.252), which may explain the differences in these studies. Moreover, men with schizophrenia in our study had a higher rate of smoking, which might also be a protective factor for cognitive function (47, 48).The mechanism might be as follows: cholinergic neurotransmission plays an important role in cognition. It has been reported that patients with schizophrenia have significant impairment of cholinergic function. The high smoking schizophrenia comorbidities observed in schizophrenics might be an attempt to compensate for cholinergic dysfunction (49). Moreover, smoking may reduce the blood concentration of some antipsychotic drugs (50).

In addition, we found statistical differences in cognitive scores between men and women with schizophrenia, but did not find an interaction between obesity and gender. Men with schizophrenia, though, showed a better cognitive state, however, after taking into account both gender and obesity, we found that the male advantage in MOCA scores, visual space and executive function, named, attention, abstract ability, and orientation disappeared. In Benjamin AM’s study, they found that there was a sex-influenced association between genetic variation at the LYPLAL1 locus and obesity-related traits (51). In Meng-Qi Chen’ s study, they found that high BMI and high waist-to-hip ratio (WHpR) have synergistic interactions with hypertension on the risk of diabetes for females (52). Moreover, Yunker AG et al. also found that female individuals and those with obesity may be particularly sensitive to disparate neural responsivity elicited by sucralose compared with sucrose consumption (53). Therefore, the interaction between obesity and gender on cognitive function needs further validation with larger sample sizes.

Finally, we explored the relationship between BMI and cognitive functions in men and women. We found that MoCA was only positively correlated with BMI in men with schizophrenia, while there was no such association in women with schizophrenia. However, Rashid NA (54) found that the indirect effect of BMI on cognition through schizophrenia was present in both sexes. Conversely, the indirect effect of cognition on BMI through schizophrenia was only found in women. In addition, Hidese et al. (55) pointed out that BMI scores were significantly negatively correlated with the Brief Assessment of Cognition in Schizophrenia (BACS) test composite score, which was in line with other research conclusions (56–58). Therefore, our findings are inconsistent. These differences might be ascribed to age and sex mismatches. In addition to that, men tend to have an earlier onset of illness while females have lower negative symptom scores (59) and are more likely to be obese. So studying these two groups separately might allow us to shed light on the relationship between gender, obesity, schizophrenia and cognition (54).

We acknowledge that there are several limitations to our research. First, this was a cross-sectional study, and it was impossible to establish causality between sex and obesity or cognition; second, all the data were collected from chronic Chinese patients who had been taking antipsychotics for a long time, and the dose and specifics of antipsychotic treatment might impact the results; third, relevant variables were not addressed, such as controlling for dose of antipsychotics and separate investigation of pre and post-menopausal women.

## Conclusions

The prevalence of obesity in elderly women with schizophrenia is higher than that in men; 2) the cognitive function of elderly women with schizophrenia is worse than that of men; 3) In this study, BMI is positively correlated with cognitive scores only in the men. In summary, there are many factors leading to cognitive decline in elderly patients with schizophrenia, among which are body mass index and gender.

## Data Availability Statement

The raw data supporting the conclusions of this article will be made available by the authors, without undue reservation.

## Ethics Statement

The Research Ethical Committee of the Affiliated Mental Health Center of Shanghai Jiaotong University School of Medicine approved the study protocol. The patients/participants provided their written informed consent to participate in this study.

## Author Contributions

WL and SL contributed to the concept and design of the study. LY acquired the data. YF and SX analysed the data and drafted the manuscript. All authors made a significant contribution to the work reported, whether that is in the conception, study design, execution, acquisition of data, analysis and interpretation, or in all these areas; took part in drafting, revising or critically reviewing the article; gave final approval of the version to be published; have agreed on the journal to which the article has been submitted; and agree to be accountable for all aspects of the work.

## Funding

This work was supported by grants from “National Key R&D Program of China” 2018YFC2002302, the Clinical Research Centre Project of Shanghai Mental Health Center (CRC2017ZD02), Shanghai Clinical Research Center for Mental Health (19MC1911100); the Cultivation of Multidisciplinary Interdisciplinary Project in Shanghai Jiaotong University (YG2019QNA10), curriculum reform of the Medical College of Shanghai Jiaotong University, the Feixiang Program of Shanghai Mental Health Center (2020-FX-03), and Youth Scientific Research Project of Shanghai Municipal Commission of Health and Family Planning (20184Y0298).

## Conflict of Interest

The authors declare that the research was conducted in the absence of any commercial or financial relationships that could be construed as a potential conflict of interest.

The reviewer MHX declared a shared affiliation with the author(s) HA to the handling editor at the time of review.

## Publisher’s Note

All claims expressed in this article are solely those of the authors and do not necessarily represent those of their affiliated organizations, or those of the publisher, the editors and the reviewers. Any product that may be evaluated in this article, or claim that may be made by its manufacturer, is not guaranteed or endorsed by the publisher.

## Abbreviations

MoCA, Montreal Cognitive Assessment; PANSS, Positive and Negative Syndrome Scale; SDs, Standard deviations; ICD-10, International Classification of Diseases 10 diagnostic standards; BMI, Body mass index; WGOC, Chinese Working Group on Obesity in China criteria; MMSE, Mini-Mental State Examination; GDS, Geriatric Depression Scale.
